# Using fragmentation to assess degradation of forest edges in Democratic Republic of Congo

**DOI:** 10.1186/s13021-016-0054-9

**Published:** 2016-06-22

**Authors:** Aurélie C. Shapiro, Naikoa Aguilar-Amuchastegui, Patrick Hostert, Jean-François Bastin

**Affiliations:** 1 World Wide Fund for Nature Germany, Reinhardtstr 18, 10117 Berlin, Germany; 2 World Wildlife Fund-US Forest and Climate Program, 1250 24th st. NW, Washington, DC 20037 USA; 3 Geography Department, Humboldt Universität zu Berlin, Unter Den Linden 6, 10099 Berlin, Germany; 4 Integrative Research Institute On Transformations of Human-Environment Systems (IRI THESys), Humboldt Universität zu Berlin, Unter Den Linden 6, 10099 Berlin, Germany; 5 Landscape Ecology and Plant Production Systems Unit, Université Libre de Bruxelles, CP264-2, B-1050 Brussels, Belgium; 6 BIOSE Department, Gembloux Agro-Bio Tech, Université de Liège, B-5030 Gembloux, Belgium

**Keywords:** Forest degradation, REDD, Fragmentation, Biomass, Emissions, Conservation

## Abstract

**Background:**

Recent studies have shown that fragmentation is an increasing threat to global forests, which has major impacts on biodiversity and the important ecosystem services provided by forested landscapes. Several tools have been developed to evaluate global patterns of fragmentation, which have potential applications for REDD+. We study how canopy height and above ground biomass (AGB) change across several categories of forest edges determined by fragmentation analysis. We use Democratic Republic of Congo (DRC) as an example.

**Results:**

An analysis of variance of different edge widths and airborne estimated canopy height found that canopy heights were significantly different in forest edges at a distance of 100 m from the nonforest edge. Biomass was significantly different between fragmentation classes at an edge distance of 300 m. Core forest types were found to have significantly higher canopy height and greater AGB than forest edges and patches, where height and biomass decrease significantly as the level of fragmentation increases. A change analysis shows that deforestation and degradation are increasing over time and biomass loss associated with degradation account for at least one quarter of total loss. We estimate that about 80 % of primary forests are intact, which decreases 3.5 % over the 15 year study period, as primary forest is either deforested or transitioned to forest edge. While the carbon loss per hectare is lower than that of deforestation, degradation potentially affects up to three times more area than deforestation alone.

**Conclusions:**

When defining forest degradation by decreased biomass without any loss in forest area, assessing transitions of core forest to edges over time can contribute an important element to REDD+MRV systems. The estimation of changes between different forest fragmentation types and their associated biomass loss can provide an estimate of degradation carbon emission factors. Forest degradation and emissions due to fragmentation are often underestimated and should comprise an essential component of MRV systems.

## Background

Deforestation and forest degradation are global problems, significantly altering ecosystems, the services they provide, while contributing to carbon emissions and affecting regulation of global climate and terrestrial carbon storage [1–3]. International mechanisms such as the reduction of emissions from deforestation and degradation (REDD+) require complete, repeatable, conservative and transparent assessment and quantification of changes in forest biomass which emit greenhouse gases in order to mitigate impacts and develop robust measurement, reporting and verification (MRV) systems [4–7].

Deforestation is defined by a long term loss of canopy cover and area, notably a conversion to another non-forest use, which been monitored effectively over time at multiple scales effectively for tropical forests using remote sensing technologies [8–14]. In contrast, forest degradation is a more poorly understood process which involves partial canopy loss with no clear reduction in forest area, but a reduction in ecosystem services, more often described by a decrease in above ground biomass [15–19], and is the definition applied in this study. The associated decrease in carbon stock and biomass are key to forest degradation assessments with respect to climate change mitigation in the context of REDD+ and thus of essential importance for determining baseline rates of degradation, in the same manner baseline deforestation is assessed [19].

The main drivers of forest degradation are related to urban expansion, extraction of forest products for both industrial and subsistence markets and associated infrastructure and accidental or deliberate fires for small-scale clearing [20, 21]. Most remote sensing studies focusing on forest degradation are driver specific and aim to detect canopy gaps and clearings through direct approaches such as spectral mixing [22, 23], or indirect methods such as mapping roads or human settlements [24, 25] or fire monitoring [26]. Still, many nations are unable to effectively monitor forest degradation at large scale over time to meet their REDD+ goals. This is more often due to the lack of a consistent definition, few robust and transparent methods for general degradation monitoring, data deficiencies, low technical capacity and limited funding [15, 27, 28]. No accurate estimates of global degradation exist to date for the reasons stated above, yet the actual extent of degraded tropical forests and associated emissions could in fact be comparable to, or larger than actual deforestation, particularly in high forest/low deforestation (HFLD) countries [1, 19, 29–35].

Recent studies have addressed the impact of human activity on the fragmentation of forests through various analyses [36–44] possible with the increase in available forest cover data and satellite imagery [12, 45, 46]. More recently, analyses have shown that core forests are more likely to be intact, providing greater ecosystem services than those exposed to edges and fragmentation. The intact forest landscapes (IFL) approach differentiates potentially intact and degraded forests worldwide [47–49] and has determined that forests are in fact structurally different outside the hinterland area [50]. Haddad et al. [39] identified fragmented forests globally as all forests within 1 km of forest edge and assessed the long term ecological consequences, including degraded ecosystem processes and declines in species richness. Riitters et al. [38] report significant deforestation of interior core forests worldwide and the resulting transitions from core forest to edge types was shown to impact twice the area affected by deforestation alone. Chaplin-Kramer et al. [44] assessed a reduction of 25 % of forest biomass in edges [44] which shows that fragmentation may indeed be a key driver of forest degradation and often lacking from forest carbon emissions accounting.

In this study we use forest cover spatial pattern to classify several types of forest fragmentation, using the optimal edge distance for which degradation is affecting forest structure and biomass. We then identify degraded forests by their transition between core and fragmentation types and use mean AGB estimates per fragmentation class to determine the associated emissions, using the Democratic Republic of Congo as an example.

We classify primary forest into four fragmentation classes defined by pattern: core (intact forest), inner edge (or perforation), outer edge (bordering large non-forest areas) and small forest patches, derived from the methods published by Vogt et al. [51]. The method involves a series of moving window analyses and union and intersection operations which determine the edge width, connectivity and holes of data in a binary forest/non forest image [52, 53]. The derivation of multiple types of edges, notably interior and exterior edge are an improvement over buffer methods which only define forests as either intact or edge, as we conclude that different types of fragmentation are demonstrated to be fundamentally and functionally different. The interior and exterior edges are in fact differentiated by the size of neighboring non-forest or forest. This analysis enables to differentiate between the impact of a small perforation within an area of intact forest which differs from for example, the edges created by a large non-forest patch which could be encroaching field or pasture. The fragmentation analysis provides insight into different patterns or drivers of degradation at forest edges, as interior holes are likely to be less accessible by anthropogenic impacts. Equally important is the appropriate distance used to assess forest edges. We use mean canopy height and AGB estimates to address this.

Assessing transitions between fragmentation classes over time allows to identification of degraded forests by the dynamic process of degradation, supporting a simple matrix approach to forest monitoring as recommended by Bucki et al. [67]. This proxy assessment is important to identify degradation by its dynamic process, which supports monitoring of forests as dynamic systems defined by their trajectories [54]. This analysis is also useful to identify degraded areas which still meet the forest criteria and using AGB estimates to quantify the ability to provide ecosystems services, which are key functions of intact forests [55]. Here we propose to use the transition between different initial fragmentation classes in order to differentiate between primary and secondary degradation and regeneration, which demonstrates the typical pathways of forest degradation and can inform forest condition.

## Methods

### The DRC context

**Fig. 1:**
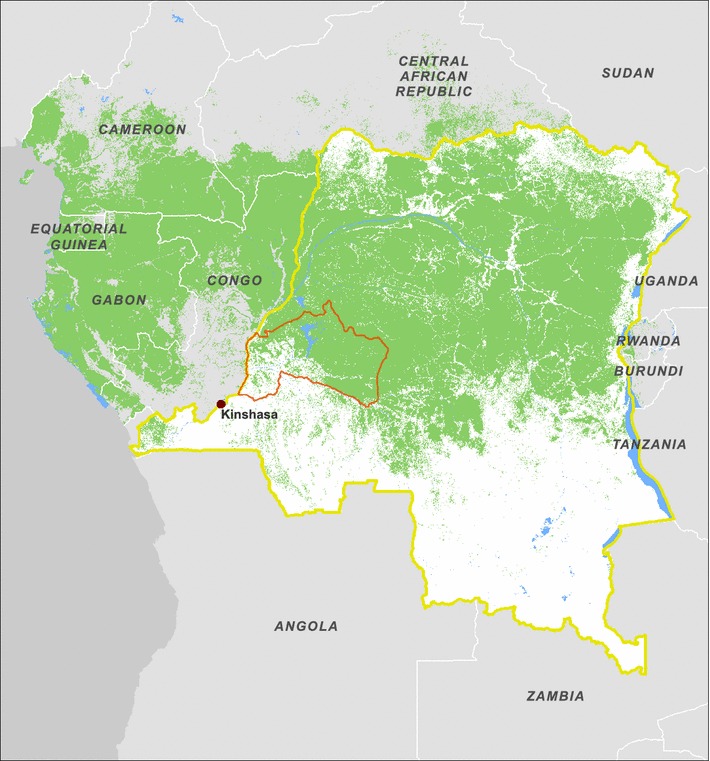
The Democratic Republic of Congo possesses the largest tract of continuous tropical forest in Africa (forest cover data from [64]). The new Mai Ndombe province region is a target site for implementation of new REDD+ activities

The Democratic Republic of Congo (DRC) possesses the largest continuous tract of remaining tropical forest in Central Africa (Fig. 1). It is known for its remarkable natural resources and high biodiversity [56, 57] while ranking nearly last on the United National Development Programme Human Development index [58]. Poor governance has allowed extensive resource exploitation such as mining, timber harvesting, charcoal production, resulting in one of the highest deforestation and degradation rates in central African countries [49]. Compared to other countries, the DRC remains a high forest/low deforestation country (HFLD) [59] and recognizes the potential for sustainable and economic development through emerging governance structures and significant engagement in the United Nations Framework Convention on Climate Change (UNFCCC) process [27, 60]. The DRC has been building up political REDD+ capacity while increasing efforts to monitor and mitigate forest loss with satellite imagery, in addition to mapping forest carbon at the national scale using airborne LiDAR and satellite imagery [61–63]. Current emissions reduction activities are focused in the Mai Ndombe region northwest of the capital, Kinshasa, which is used as a local scale test site in this study (Fig. 1).

### Datasets used

**Fig. 2:**
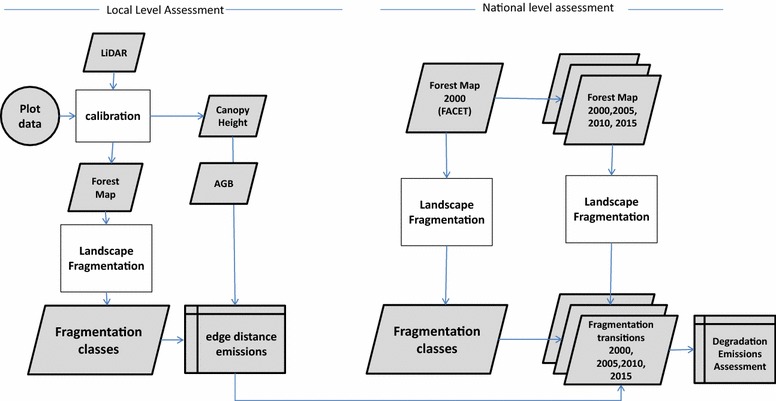
Flowchart of national scale analysis to develop fragmentation statistics and change in DRC from 2000 to 2005 and 2010 and 2015

The fragmentation algorithm was executed first at the local scale in Mai Ndombe to evaluate the effect of edge distance on biomass and canopy height available from airborne LiDAR in order to select the scale for the national analysis (Fig. 2). The local scale study encompasses LiDAR plots collected Mai Ndombe province, which are part of a collection of LiDAR collected in a stratified random manner throughout the DRC, producing an unbiased sampling of forest areas. LiDAR data were collected between October 2014 and 2015 in a series of 216 10× 2 km rectangular plots, with a mean point density of 2/m^2^. All pixels with a LiDAR mean canopy height greater than 3 m according to the national definition were classified as forest and resampled to 10 m resolution as input for the local scale fragmentation analysis. AGB estimates derived from LiDAR in Mai Ndombe were produced for the Mai Ndombe Emissions Reduction program by the University of California, Los Angeles, using the VCS VT0005 method [65] along with field data calibrated LiDAR, while the national LiDAR biomass map is still being developed for DRC.

Primary forest cover for the entire DRC for the year 2000 was derived from Landsat imagery by the University of South Dakota, the Observatoire Satellital des Forêts d’Afrique Centrale (OSFAC) and University of Maryland producing a dataset identified as Forêts d’Afrique Centrale Evaluées par Télédétection (FACET) [66]. This data is a pre-cursor to the Global Forest Cover Change product and uses similar techniques [12] producing forest maps as a resolution of 60 m and identifying primary, secondary and woodland dominated forest from 2000 to 2005 and 2010. Forest cover in the primary humid tropical forest category for 2000 was used for this analysis, as this class correlates best with moist tropical forest as defined by IPCC, while other FACET forest types mix secondary and dry forest [67]. Annual forest loss data for 2000–2014 from Global Forest Cover Change product from the University of Maryland [12] were then used to determine forest cover for 3 additional time intervals, 2005, 2010, 2015, which were combined based on the uncertainties of annual assessments of this data [68]. The gain data provided do not have a date of detection and about 20 % of gain pixels were also identified as loss, which could be due to changes in planted forests or agroforestry. In order to integrate areas of gain into the analysis, all areas of gain which overlapped with areas of loss were removed and the remaining pixels of gain were added to the final transition map to assess regeneration.

### Forest fragmentation algorithm

We used modified outputs from the Landscape Fragmentation Tool (LFT) [69] derived from the research of Vogt [52] to identify and evaluate four forest fragmentation classes: core, inner edge, outer edge and patch forest which have varying degrees of fragmentation (Table 1).

**Table 1:** Main fragmentation classes derived from Vogt et al. [51]

Fragmentation class	Description	Level of fragmentation
Core	Interior forest pixels far from forest edge	Low
Inner edge	Forest pixel on edge of small interior non-forest	↓
Outer edge	Pixels that are between forest and large non-forest areas	↓
Patch	Forest regions too small to contain core forest	High

The LFT processes a forest image using a defined edge width, which determines the edge effect distance between nonforest and intact core forest. A specific definition of edge effect for a particular locale can be used to adjust the analysis according to local information or expert knowledge on the forest of interest. We tested several window sizes and determined the statistical difference between LiDAR estimated canopy height and AGB within fragmentation classes to identify the appropriate window sizes. With smaller window sizes, a greater percentage of in the landscape is classified as core than other types; and with larger sizes a greater estimate of edge occurs [7]. The fragmentation classes produced by edge distances of 50, 100, 150, 200, 250, 300, 350 and 500 m were evaluated for statistical differences in canopy height and AGB. A set 5000 points located randomly within the LiDAR footprints in Mai Ndombe were selected to assess canopy height and estimated AGB within each fragmentation class produced with varying edge distances. The mean canopy height difference between samples in each fragmentation class was determined using an analysis of variance ANOVA for all sample points. A Tukey honest significant difference and Mann–Whitney pairwise tests for non-parametric data were performed to determine a significant of difference in mean canopy height and biomass between each fragmentation category pair. Statistics were performed using the R statistical package version 2.14.0 and Past Version 3.10 [70].

Additionally, a semivariogram analysis was used to assess heterogeneity in canopy heights to determine the best minimum mapping unit for forest cover data by estimating semivariance over progressively larger window sizes. Thus, forest cover at the national scale was rescaled to 1 ha resolution, informed from the LiDAR data analysis.

### National scale analysis

**Fig. 3:**
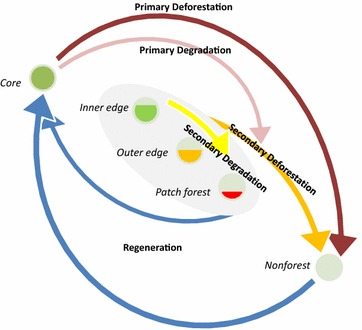
Transition pathways between forest fragmentation types, using fragmentation classes to differentiate between primary and secondary deforestation and degradation. Reverse trends (from more degraded categories towards core) are recovering forests. Forests that remain in the same class over time are named “stable”

The primary forest data were resampled to 100 m based on results from semivariography analysis of the LiDAR canopy height data. Fragmentation classes were assessed for each forest cover map and the transitions between fragmentation categories over time were identified as in Fig. 3. Mean AGB for each class of new degradation was used to provide the estimated biomass loss (emission factor) for all degradation transitions to calculate emissions from forest fragmentation at the national scale, based on a tier I stock difference approach, using biomass estimates and the area of forest cover lost at each time period [67, 71, 72].

## Results

**Fig. 4:**
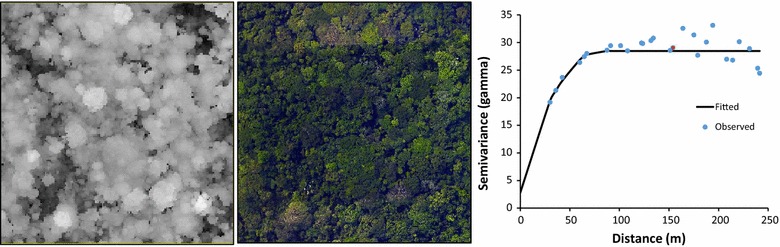
One of the 12 ha areas assessed with semivariography, showing the true-color image (*left*), mean canopy height (*center*), and corresponding semivariogram (*right*). Semivariogram symbol indicates semivariance frequency with *blue dot* indicating highest frequency at 154 m

Semivariogram spherical modeling parameters with better fit averaged in the 110 ± 7 m range, which was used as a metric to estimate the spatial dimension of forest structural heterogeneity. Thus, a minimum mapping unit (MMU) of 100 m was used for mapping forest cover at national scale Fig. 4.

**Fig. 5:**
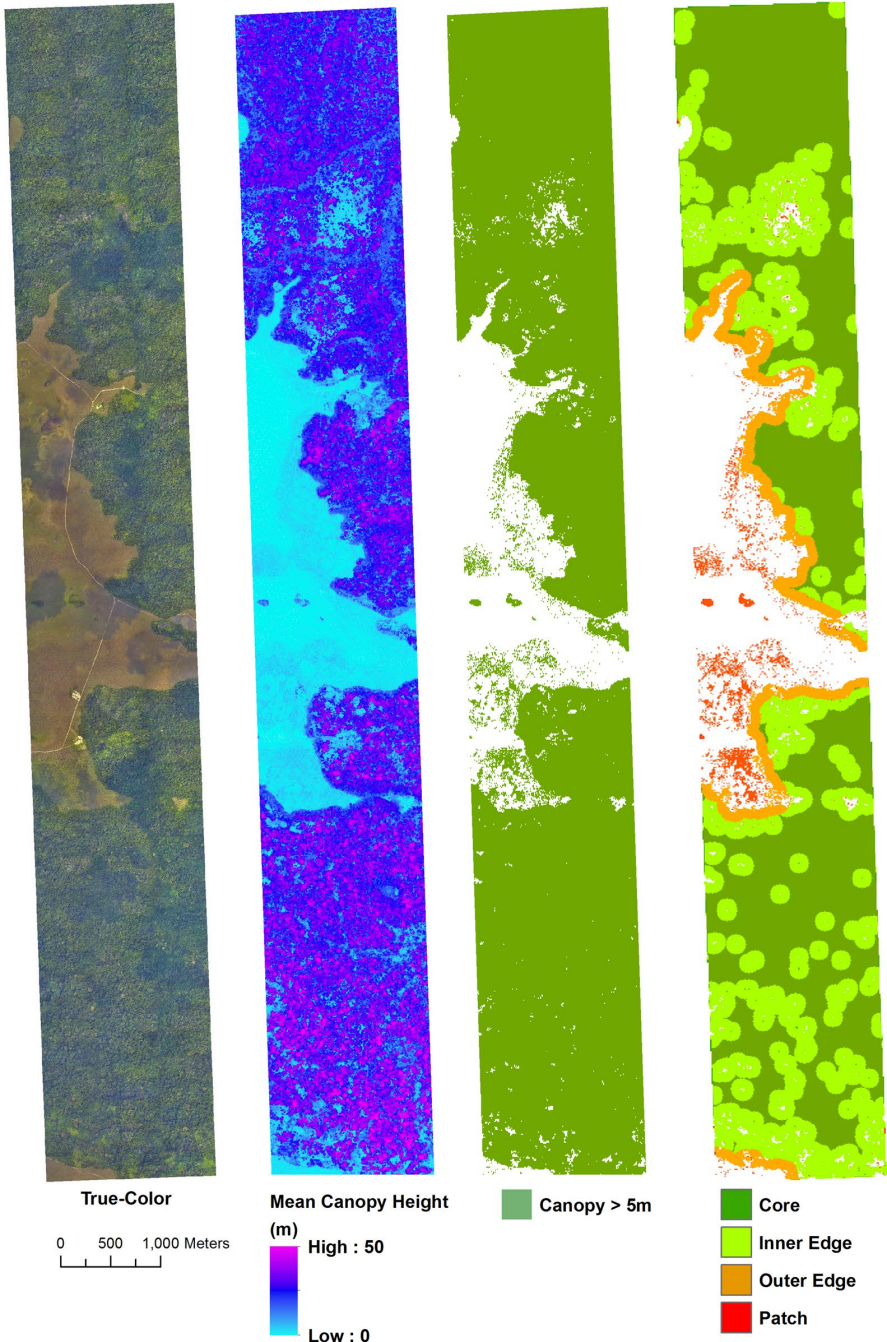
Sample 10 km x 2 km LiDAR plot used in the local scale analysis. From *left* to *right*: 10 cm aerial photo; mean canopy height from LiDAR returns at 15 m resolution; forest/non-forest map obtained by filtering mean canopy heights below 5 m (per country forest definition); Fragmentation classification

**Fig. 6:**
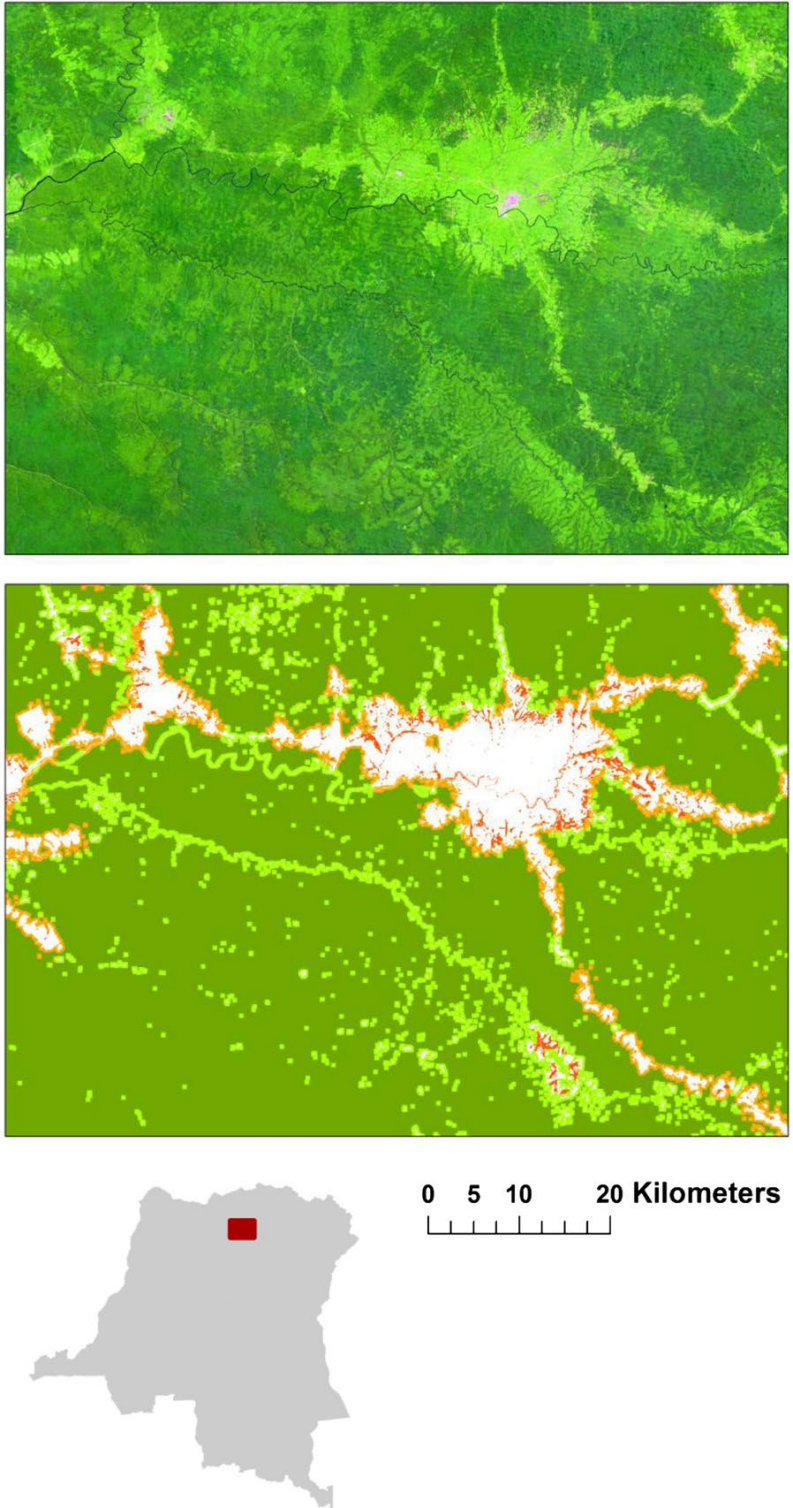
Example of fragmentation assessment in northern DRC. *Top*: True-color *Red*, *Green*, *Blue* Landsat 2005–2010 composite from Forêts d’Afrique Centrale Evalués par Télédétection (FACET) [66]; *Bottom*: Forest fragmentation is calculated for 2005 (*green*: core forest, *light* *green*: inner edge; *orange*: outer edge; *red*: patch)

Forest fragmentation classes generated for the high resolution/small spatial scale analysis from LIDAR data collected in 2014 are shown in Fig. 5, with canopy height, forest cover and AGB derived from airborne LiDAR acquired during the study period. A subset of the FACET landsat data and derived fragmentation classes show how forest edges occur around villages (Fig. 6). Forest heights were highest in core forest areas and decrease into significantly lower averages as fragmentation increased.

Mean canopy height within forest fragmentation classes derived from LiDAR heights were found to be significantly different at all scales in the ANOVA, however, the non-parametric tests for the differences between paired categories varied. Only at the scale of 100 m was the difference in canopy height between all fragmentation classes significant (Mann–Whitney p ≪ 0.005).

**Fig. 7:**
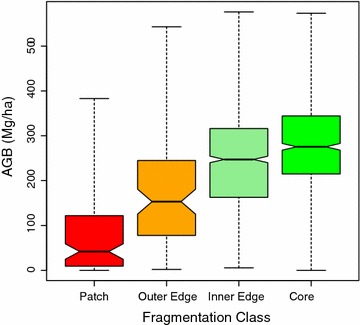
Distribution of AGB estimated from airborne LiDAR for fragmentation classes derived with an edge distance of 300 m; model p ≪ 0.005

AGB estimates showed differences on a different spatial scale than canopy height. While all edge distances showed significant differences, only an edge distance of 300 m produced significantly different differences of AGB between each fragmentation class pair (Mann–Whitney p ≪ 0.005) (Fig. 7).

### National scale temporal changes

**Fig. 8:**
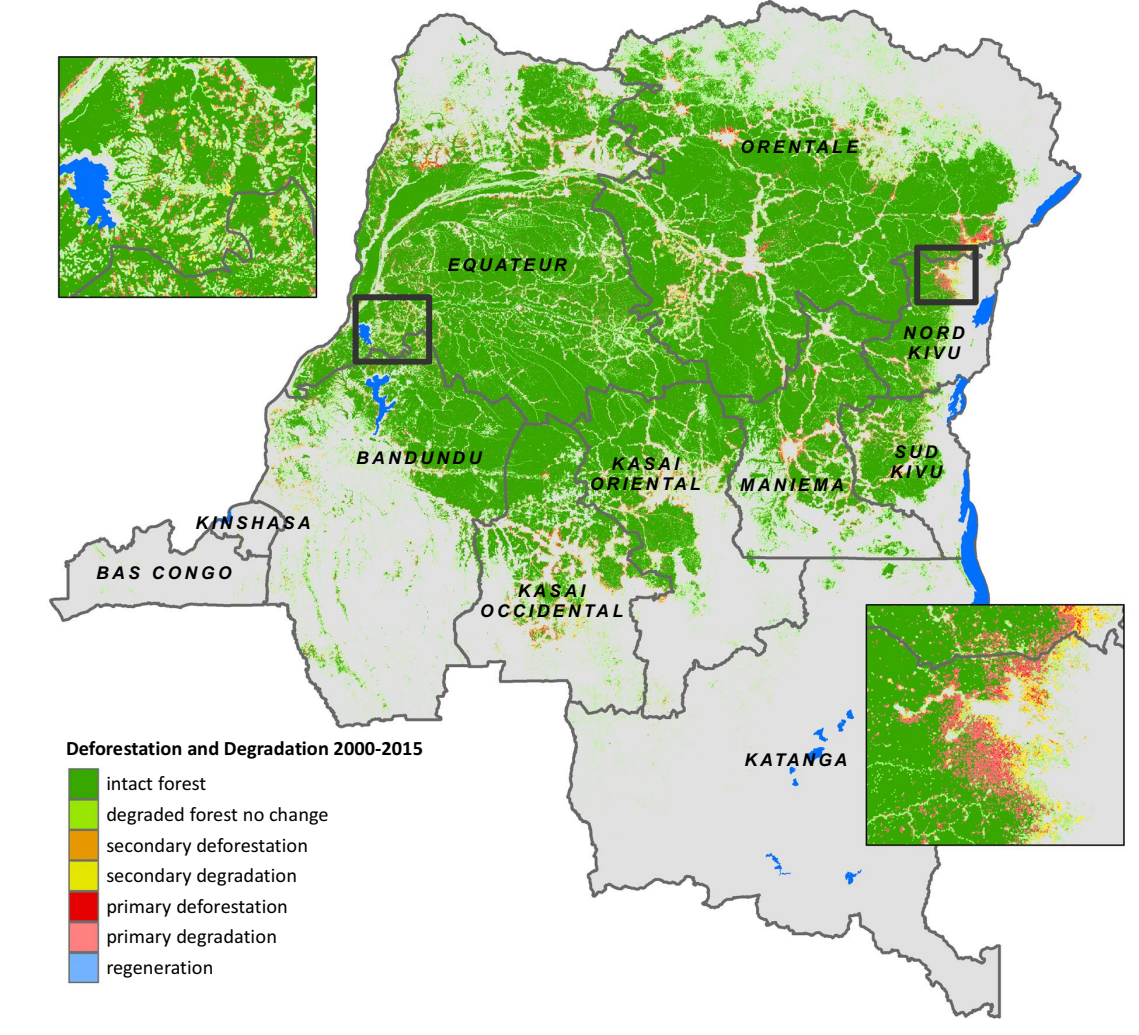
Forest fragmentation change from 2000–2010, showing transitions between fragmentation classes. *Insets* show areas of significant degradation in North Kivu Province around Beni, and more diffuse degraded forest *edges* in the forest mosaic of Mai Ndombe around Mbandaka. Primary and secondary degradation appear to be concentrated around cities and access routes. The largest areas of forest undergoing degradation are in North Kivu province, with the most fragmented forests occurring in the transition to savanna landscapes in western DRC. Small recovery areas were observed where forest patterns areas change from outer edge to inner edge (less than 1000 km^2^ overall, not visible at the *national scale map*) which are due to consolidation of forest areas into more uniform shapes

Overall forest cover decreases over the study period. Core forest decreases 3.5 % over the study period, inner and outer edge increase and patch forest remains about the same (Table 2). The transitions between fragmentation classes on a 1 ha pixel basis from 2000–2005–2010–2015 are reported in Table 3 and mapped for the entire DRC primary forest belt in Fig. 8. Core forest is most often transitioned to inner edge and outer edge is more often deforested than other fragmentation classes.

**Table 2:** Total core and degraded forest types for 2000, 2005, 2010 and 2015, with percent of total forest area. Forest gain is included to the 2015 forest cover

Fragmentation class	2000		2005		2010		2015	
	Km^2^	% of forest	Km^2^	% of forest	Km^2^	% of forest	Km^2^	% of forest
Core	827,824	79.4	814,319	78.4	798,604	77.4	775,755	75.9
Inner edge	81,785	7.8	90,946	8.8	98,170	9.5	108,012	10.6
Outer edge	97,399	9.3	97,483	9.4	97,953	9.5	98,675	9.7
Patch forest	35,156	3.4	35,584	3.4	36,629	3.6	39,006	3.8
Total forest	1,042,164		1,038,332		1,031,356		1,021,447

**Table 3:** Transition matrices estimating change between fragmentation classes in km^2^ from 2000 to 2005 (top) and from 2005 to 2010 (middle) and from 2010 to 2015 (bottom)

Transition to (2005)						
Transition from (2000)	Core	Inner edge	Outer edge	Patch	Nonforest	Total
Core	814,298	11,339	1425	11	751	827,824
Inner edge	0	79,574	1348	54	807	81,783
Outer edge			94,698	979	1670	97,347
Patch				34,539	604	35,143
Nonforest					1,305,354	

Transition to (2010)						
Transition from (2005)	Core	Inner edge	Outer edge	Patch	Nonforest	Total
Core	798,605	12,405	2196	40	1073	814,319
Inner edge		85,764	3100	216	1865	90,945
Outer edge			92,656	1874	2952	97,482
Patch				85,764	1085	86,849
Nonforest					1,309,186	

Transition to (2015)						
Transition from (2010)	Core	Inner edge	Outer edge	Patch	Nonforest	Total
Core	775,753	17,833	3087	61	1870	798,604
Inner edge		90,176	4765	460	2765	98,166
Outer edge			90,822	3065	4063	97,950
Patch				35,418	1209	36,627
Nonforest					1,316,162

The largest transition in fragmentation classes observed from 2000 to 2015 was primary degradation, notably in the transition from core forest to inner edges, followed by degradation of inner to outer edges. The most significant observation at the national scale is that overall area of degradation increased nearly by 50 % in the time period and when associated with biomass estimates, resulted in a quarter of total forest related emissions (Table 4).

**Table 4:** Contribution of deforestation and degradation of primary forests to total forest emissions

	2000–2005		2005–2010		2010–2015	
	Def.	Deg.	Def.	Deg.	Def.	Deg.
Area (km^2^)	3832	15,157	6975	19,832	9908	29,272
Biomass loss (MgC)	63,709,538	33,235,831	116,081,439	39,374,186	168,515,864	56,426,709
Tons CO_2_ equivalent	233,176,909	121,643,141	424,858,067	144,109,521	616,768,062	206,521,755
% of total CO_2_ emissions	65.7	34.3	74.7	25.3	74.9	25.1

Inner edge increases much larger than the other classes, more than 40,000 km^2^. The total degraded area increases from 2000 to 2015, with a much greater increase in the 2010 to 2015 time period. Primary forest loss increases over time and was highest in the 2010–2015 time period than the previous 5 year intervals.

Area calculations show increases in degradation in 2005–2010, 2010–2015 compared with the first 5 year span, with the greatest transition occurring between core and edge classes. This results in more than double the area affected by degradation as deforestation in the second 5 year span; and a far greater proportion of associated emissions. There is a larger increase in inner edge throughout the analysis. Several examples of this have been found, indicating that clearings may be increasingly further in the forest. Of the total 6295 km^2^ of primary deforestation, 2603 km^2^, or nearly a third transition to a degraded state before deforestation. As for secondary deforestation, which was overall greater than primary deforestation (14,420 km^2^), only 834 km^2^ transition to a degraded state before deforestation.

### Emissions estimates

Table 4 shows the biomass losses estimated for the 5 year intervals from 2000 to 2015. Deforestation is steadily increasing, as is degradation. The overall area affected by degradation is shown to be much larger than that affected by deforestation, however, emission per hectare are lower, thus degradation contributes to a lower proportion of emissions, as most primary degradation is within inner edge and results in lower emissions.

## Discussion

Bucki et al. [67] recommend the development of a matrix approach (i.e. the gross calculation of transitions from intact to non-intact forest lands) for forest monitoring to help countries with limited resources monitor and reduce emissions from degradation. Indirect approaches, including the use of proxies applied over time may be useful and accurate for estimating areas of forest degradation and decreased carbon stocks, especially when direct detection by high resolution satellite imagery is problematic due to data costs, presence of clouds, or the area of interest is large [73]. The assessment of forest fragmentation in the temporal domain by the detection of new forest edges can be useful in this respect, because forest edges have greater human access and associated anthropogenic effects and have been shown to have significantly less biomass, increased tree mortality and lower biodiversity, all characteristics of degradation [39, 44, 74–77]. Regardless of human intervention, forest edges will always have different properties and structure associated with edge environments, but the detection of new edges occurring next to deforested areas is essential to differentiating degradation from secondary forests, which may be stable, or regenerating. In addition, as nearly one-third of primary degradation ends up as deforestation eventually, the fragmentation analysis presents an important assessment of potential future deforestation. A spatial assessment of edge and core forests and their transitions allow the assessment of forest dynamics, which should constitute a good proxy for forest degradation [38].

This research has shown how fragmentation classes defined by forest patterns have significantly different canopy height and biomass allowing their potential use as strata to discern or monitor forest uses or biomass dynamics required for national forest inventories [78], when other information on land use may be lacking [78]. Using forest cover maps from multiple time periods and deriving the associated transitions between fragmentation classes over time can be used to derive major forest cover changes and dynamics, such as primary and secondary deforestation, primary and secondary degradation and regeneration which provide more information on forest dynamics and uses than simply estimating forest cover [38, 54, 55]. Most importantly we show here that degradation at forest edges actually affects more area than deforestation. Combining this information with available AGB data allows for the estimation of biomass loss from these changes which is one of the required carbon pools for REDD+ reporting.

The selection of edge distance is important to determine before the analysis and affects the estimation of area defined as degraded edge. Canopy height was shown to be different within fragmentation classes, which is evidence of structural differences at forest edges. However if we look at forest height alone, we see that secondary forests can quickly reach similar heights as intact forests, which complicates optical remote sensing of degradation. Thus, biomass is the important measure and essential to defining forest degradation. The resolution of the biomass estimates is also important as it would be difficult to discern edge effects at the sub-pixel scale, for this reason Chaplin-Kramer et al. [44] suggest an edge distance that is much larger. Pelletier et al. [7] however showed that edge distance is actually the lowest source of uncertainty compared to other factors when estimating emissions. Here we suggest a window size which effectively stratifies forests based on the available accurate estimates of biomass.

The fragmentation analysis employed is straightforward, repeatable and easily executed. A simple proxy indicator does not necessarily mean higher uncertainty, and this can be informed by field data, which are always needed to improve algorithms to assess edge forest structure and transitions, also for biodiversity indices to inform comprehensive biodiversity safeguard monitoring. Additionally, determination of appropriate analysis window size and resolution to define minimum mapping units (MMUs) by applying geospatial statistics approaches such as semivariography of carbon estimates or field data can inform the most suitable resolution for forest and biomass mapping.

**Fig. 9:**
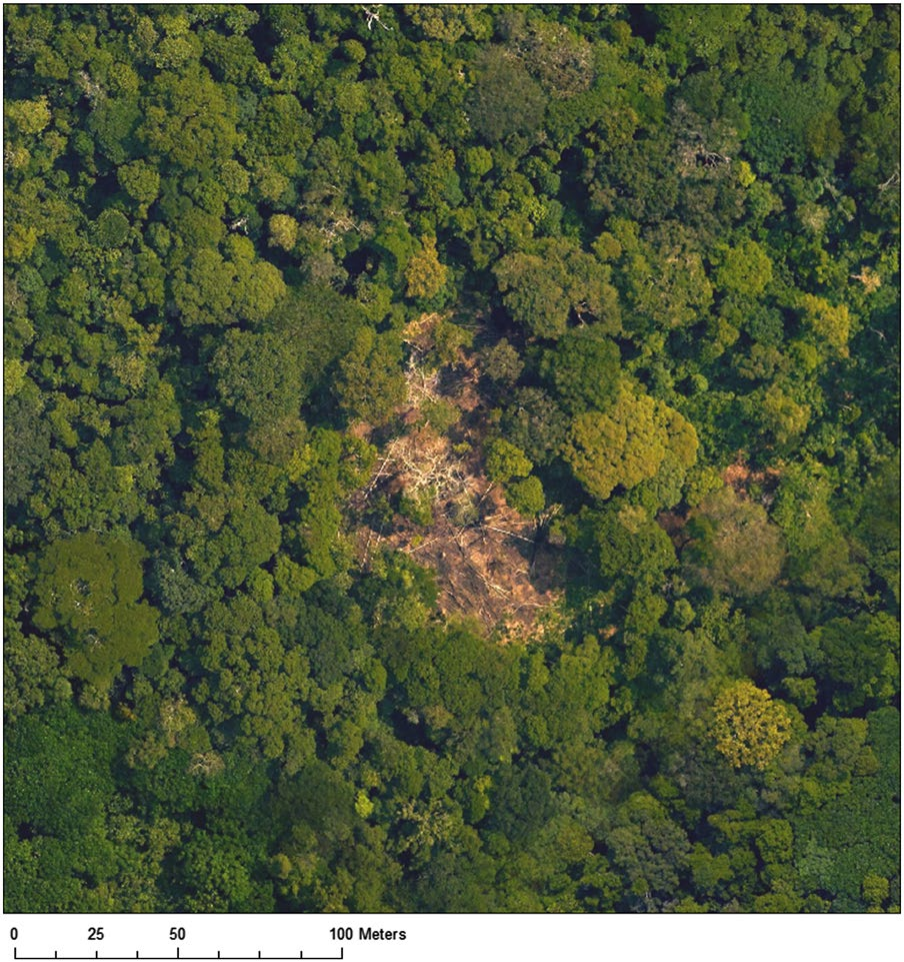
An example of a conversion of core forest to a perforation with inner edge

Our results support the findings of Zhuravleva et al. [49] and Molinario et al. [43]. Both studies estimate a greater area of forest that is affected by degradation than deforestation, with an increase in degradation observed in 2005 to 2010, compared to the previous 5 years. However, the areal estimates are different and difficult to compare directly, because Zhuravleva et al. combined degradation with deforestation, estimating that 40 % of primary forests are degraded. On the other hand, Molinario et al., present very similar results for changes in fragmentation, but they do not specifically refer to degradation. Zhuravleva et al. did observe a decrease in fragmentation rate in the 2005–2010 time period than 5 years prior, while we observe an increase in the second 5 year span, due to the fact that we assess changes between successively degraded classes as degradation, whereas with IFL degraded forests remain in the same class and thus secondary degradation is not entirely accounted for. This is an important distinction, as degradation is a process, resulting in various levels of degradation and further degradation of secondary forests can still result in further loss of ecosystem services and emissions. Small perforations within intact forest have been shown to increase. These create interior edges which have a higher AGB than outer edges, which demonstrate how fragmentation and associated degradation can vary in degree [40, 74]. Many examples of this phenomenon have been observed (Fig. 9), showing that people may be entering deeper in the forest to either clear forests with better timber or perhaps to evade detection.

Given the significant difference in biomass between fragmentation classes and the observed transitions and associated emissions, this method shows a distinct advantage over other approaches which lump degradation into one class, define degradation at one point in time, or identify fragmentation as deforestation or shifting cultivation [39, 43, 44, 50]. The assessments which assess only intact and edge forest may ignore the different possible degraded states and prevent differentiating forests which are being degraded from those which may be regenerating. It is clear in this example that forests are experiencing several degraded states in the degradation, deforestation or regeneration process and the forest fragmentation method applied to subsequent forests maps allows one to distinguish, or even stratify forests by these transitions, which is an important element for monitoring of dynamic forest systems [54].

It is also important to consider the aspects of spatial scale, especially given the common misconception that higher resolution is necessarily better. The aggregation of data to a 1 ha MMU for canopy height, and 300 m scale for AGB is an important consideration here, as studies have shown how forest biomass estimates change with scale [79]. Degradation has a spatial dimension which must be considered at a scale of the forest, rather than trees and in this case, biomass is being used as the definition for degradation. The difference in DRC degradation estimates between other published results demonstrate the importance of a universal definition of degradation including the element of spatial scale.

### Sources of uncertainty

The main limiting factor to this method is ultimately the quality of the forest cover map. In this example we use data from FACET [66], which was considered best available at the time and considered a benchmark product for DRC and was derived specifically for DRC. Higher resolution, global algorithms which use temporal mosaics to reduce cloud cover may contribute to improve the quality of the analyses [12], however this annual data has been found to suffer from low accuracy in some key locations [68, 80], which is why the Google Forest Cover change products were merged to 5 year intervals. The element of forest gain may be underrepresented here, due to the lack of date associated with this information. As a result, regeneration overall was found to be negligible compared to other transitions. Lastly, persistent forests, which may act as carbon sinks and potentially offset carbon emissions [81] are another unknown contribution to the carbon accounting in DRC.

There are several potential sources of error at many scales, particularly when measuring proxies which need to be considered. Errors from LiDAR derived estimates are identified as outliers and easily corrected. However, there remain uncertainties, in both the LiDAR derived biomass and the global biomass map. In the LiDAR data, errors were found to be similar to errors in field plots, which can be as high as 20 %. The global biomass map is accompanied by an uncertainty map, which can be used to estimate confidence intervals in emissions estimates. Pelletier et al. [7] provided a thorough review of the large potential errors and uncertainties in estimating emissions using the matrix method in Panama. Of particular attention are the sensitivities and uncertainties related to buffer width in determining area of degradation and the biomass estimates. The latter will be significantly reduced in DRC with the production of a new national LIDAR-derived biomass map with a resolution of 1 ha, which will allow detection of biomass changes in more detail and more conservative estimates of degradation. The authors also recommend increasing tier level with more localized information, accuracy assessment of proxy results and adhering to principles of consistency and conservativeness which should also apply for DRC and including a critical assessment of model uncertainties and how to apply them conservatively and consistently over time.

### Biodiversity safeguards

Carbon emissions aside, what is potentially a more useful application of forest fragmentation analysis is the impacts of increased forest degradation on habitats. As the additional requirements to operationalize biodiversity safeguards are implemented, this degradation proxy can be used in combination with biodiversity information to assess ecosystem services and risks to biodiversity, which are based on the principles of landscape ecology, which have demonstrated important relationships between habitat area, quality, with biodiversity. The effects of fragmentation have been shown to critically impair the ability of an ecosystem to provide viable habitat through decreased area, increased isolation and edges [39]. These are propagated throughout the ecosystem, affecting species richness, persistence, community composition among other effects and along with an increase in anthropogenic access can provide a solid basis to use fragmentation to evaluate essential habitat indicators for biodiversity safeguards in REDD+ projects. An intact forest can then support not only increased biomass for climate mitigation, but the ecosystem services that local communities require—pollination, non-timber forest products, water regulation etc.…which will improve livelihoods and reduce pressure to deforest and degrade forest resources.

## Conclusion

As global deforestation and degradation increase, there is an even greater need for accurate data for assessing forest cover change and associated emissions [82]. The results of this forest pattern analysis show extensive forest fragmentation and degradation of forest edges in DRC, which is greater than the area affected by deforestation alone. This can result in adverse and long-lasting effects on biodiversity and ecosystem services [39]. Many attempts to develop sub-jurisdictional REDD+ programs and define baselines for relative emissions levels have opted to avoid estimates or calculations of unplanned degradation from their baselines and reductions targets. This research demonstrates a transparent, repeatable and simple method for including degradation in MRV systems for a matrix method approach to forest monitoring, using any available forest cover map, which should support countries with limited resources and vast forests [67].

This analysis has allowed a more detailed look at a fragmentation algorithm and the correlation between degraded forests and above ground biomass. Degradation is an especially relevant and important aspect of emissions reduction and conservation activities and when little information is available for mapping forest condition, this proxy can serve as a cost-effective tool in assessing degradation over time. Using forest cover maps derived for different years, the analysis enables one to assess reference condition, change over time and the trajectory which is a required component for monitoring degradation for REDD+ [15]. The benefit of the approach proposed here is the ability to separate degrading or regenerating forests by their trajectories between degraded classes. This helps assess potential hotspots of degradation, as well as the existence of secondary forest carbon sinks to drive management interventions to promote regeneration.

The effect of carbon map resolution may have an important role here. The DRC is currently mapping national forest carbon stocks via integrated field, satellite and airborne LiDAR, an initiative funded by the German Ministry of Environment and Nuclear Safety (BMU) International Climate Initiative and the KFW Development Bank [63]. This work has included the collection of more than 400,000 ha of airborne LiDAR throughout the country, enabling a more detailed look at canopy structure, biomass, degradation and producing better estimates of forest carbon in areas with little available data to data, or areas with particularly high error. This data will greatly improve access to reliable and unbiased biomass data.

Future steps for quantification of forest degradation will include an assessment of causes, notably from the addition of information on drivers of degradation [62] and higher resolution biomass. This will enable correlation of auxiliary data to model degradation based on human factors such as infrastructure, fire, distance to population centers which can support the development of future baselines of forest degradation for REDD+ in DRC.
